# Anatomical Study of the Angular Branch of the Thoracodorsal Artery and the Lateral Border of the Scapula With Application to Reconstructive Surgery

**DOI:** 10.7759/cureus.101559

**Published:** 2026-01-14

**Authors:** Emma R Lesser, Rarinthorn Samrid, Jacie Pujol, Julia Bishop, Jared Rosbrugh, Kathleen C Bubb, Kazzara T Raeburn, Blair M Barton, Joe Iwanaga, R. Shane Tubbs

**Affiliations:** 1 Neurosurgery, Clinical Neuroscience Research Center, Tulane University School of Medicine, New Orleans, USA; 2 Department of Anatomy, Khon Kaen University, Khon Kaen, THA; 3 Neurosurgery and Structural and Cellular Biology, Tulane University School of Medicine, New Orleans, USA; 4 Anatomical Sciences, Weill Cornell Medicine, New York, USA; 5 Anatomical Sciences, St. George's University, St. George's, GRD; 6 Otorhinolaryngology, Ochsner Health, New Orleans, USA; 7 Neurosurgery, Ochsner Health, New Orleans, USA

**Keywords:** anatomy, cadaver, reconstructive surgery, scapula, thoracodorsal artery

## Abstract

The angular artery is a branch of the thoracodorsal artery, which is the terminal branch of the subscapular artery and is known to supply the inferior angle of the scapula. The lack of detailed anatomical research and clinical outcome studies hinders the ability to fully exploit the use of this artery and the scapula it supplies. Addressing such knowledge gaps through focused anatomical studies and large-scale clinical trials will enhance the understanding and utilization of the angular branch in surgical practice, ultimately improving patient outcomes. Seven sides (five on the left sides and two on the right sides) from six embalmed cadavers were used in this study. The subscapular artery arising from the axillary artery was identified supine with the arm extended. The thoracodorsal artery was dissected under the surgical microscope to ascertain where it supplied the bone. Macroscopic and microscopic observation was conducted. The angular branch had a consistent course traveling inferiorly on the lateral border of the scapula on six sides out of seven. In three specimens, the angular branch was found to have a clear continuation that supplied the inferior border. In one specimen, a branch wrapped around the lateral border of the scapula, traveling toward its medial aspect. The lateral border was thicker than the medial border on all sides. Histological observation of the lateral border demonstrated blood cells within the trabeculae. We confirmed that the angular branch of the thoracodorsal artery supplies the lateral border and inferior angle of the scapula. This fundamental anatomical knowledge will help plan future reconstructive surgeries.

## Introduction

The angular artery is a branch of the thoracodorsal artery and is known to supply the inferior angle of the scapula [1,2]. The scapula is surrounded by of rich anastomoses that often have the potential to provide surgical applications for reconstructive surgeries in the limbs and the head and neck [3,4].

The lateral border and inferior angle of the scapula can be harvested for reconstructive surgery for many reasons, including its redundant vascular supply from the subscapular anastomosis, the suitability of the subscapular arteries as a pedicle given the vessel size, and the presence of cortical bone for structural support [5]. The inferolateral scapula with the attached angular branch can be used for maxillofacial reconstruction, for example, following resection of tumors [6]. However, the lack of detailed anatomical research and clinical outcome studies hinders the ability to fully exploit the use of this artery and the scapula it supplies. Addressing such knowledge gaps through focused anatomical studies and large-scale clinical trials will enhance the understanding and utilization of the angular branch in surgical practice, ultimately improving patient outcomes.

Therefore, this study aimed to further define the end course of the angular branch of the thoracodorsal artery and assess its role in vascularizing the lateral border and inferior angle of the scapula. The findings presented in this study are hoped to be applied to reconstructive surgery in the head and neck.

## Materials and methods

Seven sides (five on the left sides and two on the right sides) from six embalmed cadavers were used in this study. The cadavers were derived from two females and four males. The mean age at the time of death was 73.8 years (range 61 to 96 years). The subscapular artery arising from the axillary artery was identified supine with the arm extended. It was followed as it split into the circumflex scapular and thoracodorsal arteries. The thoracodorsal artery was then dissected to determine if the angular branch traveling into the inferior angle of the scapula was present. If this branch were intact, the specimen would have been recorded for harvest. The scapulae and associated thoracodorsal arteries were harvested. Branches of the angular artery were carefully followed and dissected further under the surgical microscope (Zeiss, Oberkochen, Germany) to ascertain where they supplied the bone.

**Figure 1:**
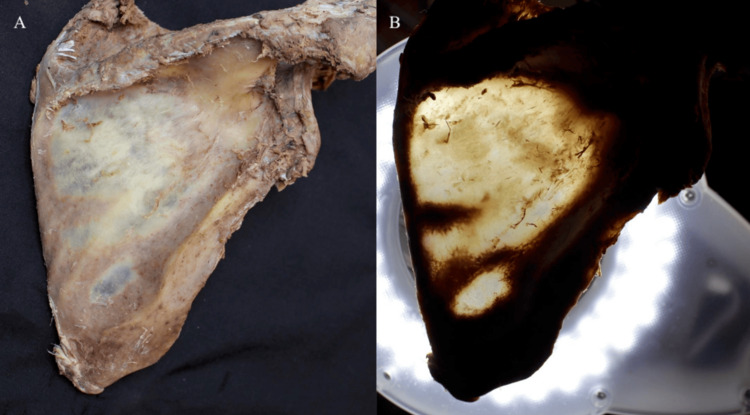
(A) The soft tissue on the scapula was removed, and (B) the thickness of the bone was measured using a transillumination technique.

**Figure 2:**
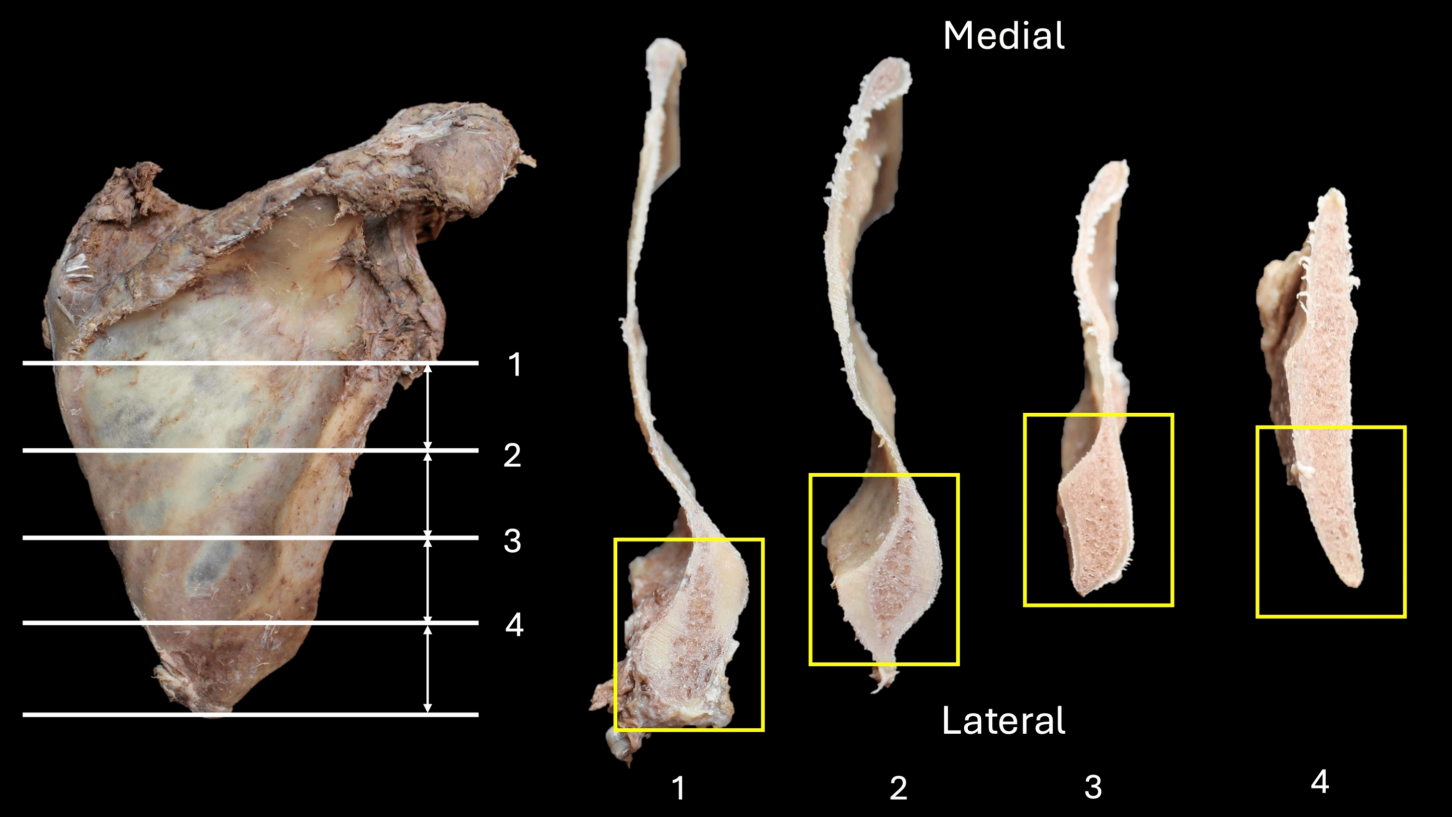
The scapulae are cut horizontally using the bone saw in four slices of equal distance. Yellow rectangular areas are observed with histology.

The distance from the inferior angle of the scapula to the osseous entry point of the angular artery was measured, and the average length was obtained. After the measurements, the soft tissue on the scapula was removed. A transillumination technique was used to visualize the thickness of the bone (Figure 1) [7]. The scapulae were then cut horizontally using a bone saw in four slices (Figure 2). The initial cut was made between the root of the spine to the inferior border of the glenoid fossa (line 1, Figure 2). Then, the distance from line 1 to the apex of the inferior angle of the scapula was divided into four equal lengths and cut into four pieces. In the axial plane, the superior surface of the lateral border of the scapula was observed. Finally, the lateral border was harvested and embedded in paraffin. A microtome was used to cut these samples into 5 µm slices, stained with Masson’s trichrome, and observed under a light microscope.

The present study was performed in accordance with the requirements of the Declaration of Helsinki (64th WMA General Assembly, Fortaleza, Brazil, October 2013). The authors state that every effort was made to follow all local and international ethical guidelines and laws that pertain to the use of human cadaveric donors in anatomical research [8].

## Results

**Figure 3:**
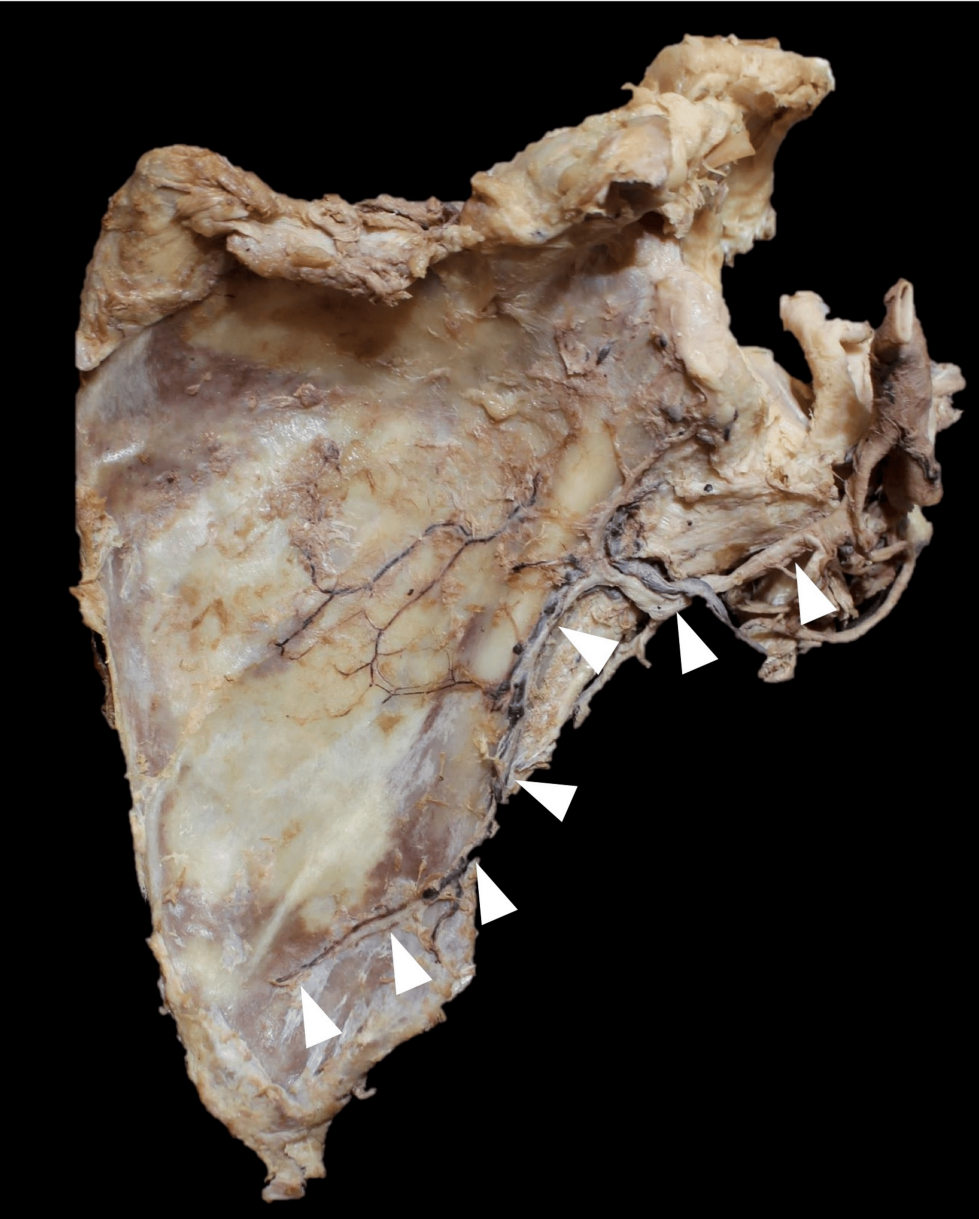
The angular branch traveling inferiorly on the lateral border of the scapula (arrowheads).

The angular branch had a consistent course traveling inferiorly on the lateral border of the scapula on six sides out of seven (Figure 3). The number of the angular branch was two on four sides and one on two sides. The vertical and horizontal mean distance from the inferior angle of the scapula to the osseous entry point was 32.7 mm (range from 22.5 to 46.9 mm) and 12.0 mm (range from 2.4 to 24.5 mm), respectively. In three specimens, the angular branch was found to have a clear continuation that supplied the inferior border. In one specimen, a branch wrapped around the lateral border of the scapula, traveling toward its medial aspect.

**Figure 4:**
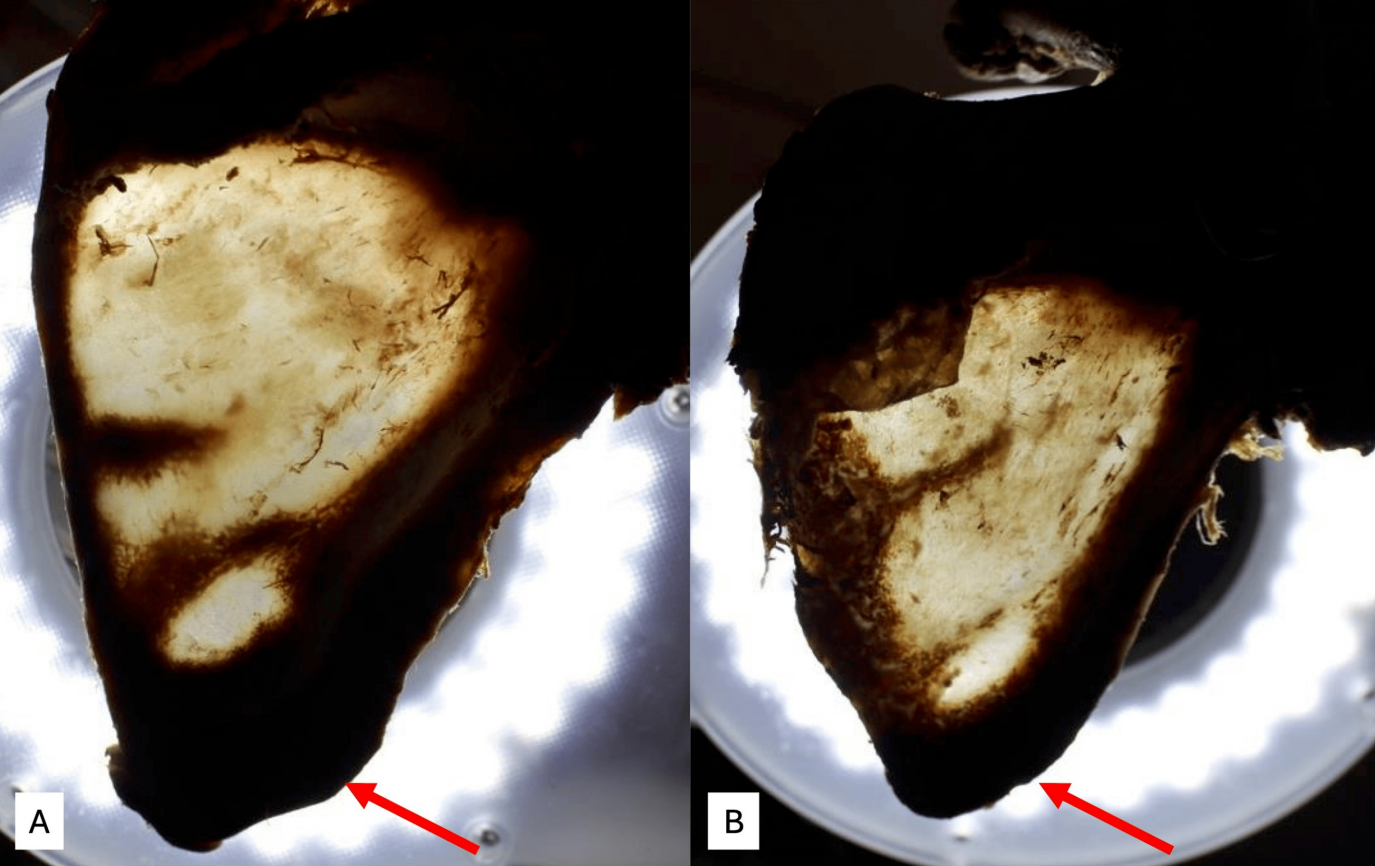
The transillumination technique demonstrated that the lateral border and inferior angle were thick and dark in all specimens. (A) A case with the angular branch reaching the inferior angle (arrow). (B) A case with the angular branch not reaching the inferior angle (arrow).

**Figure 5:**
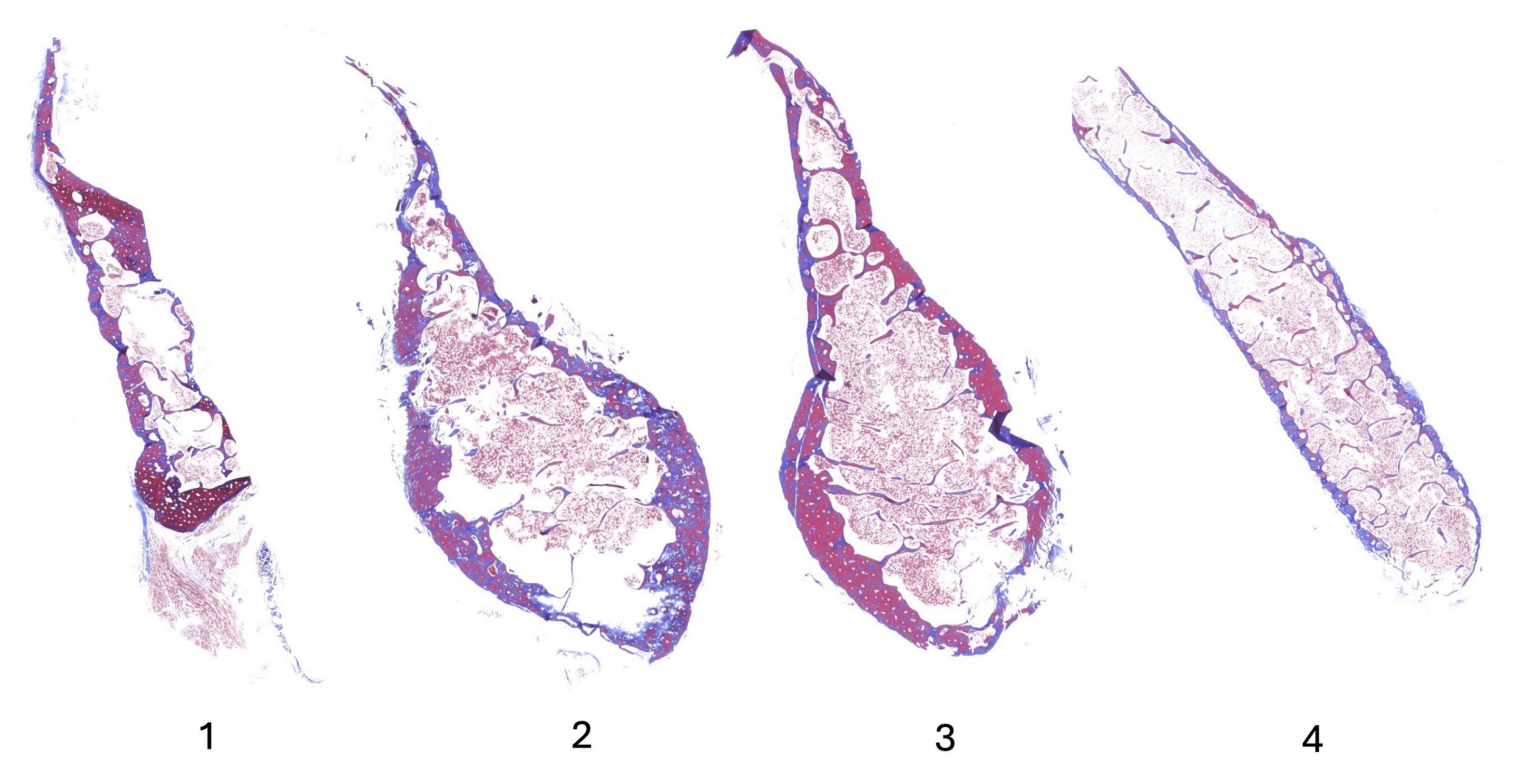
Histological observation of the lateral border (rectangular area shown in Figure 2) demonstrates blood cells within the trabeculae.

The transillumination technique demonstrated that the lateral border and inferior angle were thick in all specimens. In cases where the angular branch was confirmed to reach the inferior angle, the inferior angle looked, more or less, thicker than in the case with a shorter angular branch, which did not reach the inferior angle (Figure 4). The lateral border was thicker than the medial border on all sides. The horizontally cut sample showed consistent findings. Histological observation of the lateral border demonstrated blood cells within the trabeculae (Figure 5).

## Discussion

The scapular dimensions were measured to clarify the characteristics of the scapular borders. The superior border is almost horizontal or gently sloping downward from the superomedial angle toward the root of the coracoid process. The medial border lies between the superior and inferior angles, while the lateral border extends from the inferior angle up to the lower margin of the glenoid fossa [9].

The present study has shown that the lateral border of the scapula can be thicker and have a richer blood supply than the medial border, which is supplied by the angular branch of the thoracodorsal artery [10]. In addition, in the study on maxillary reconstruction, the distance of the lateral scapula bone harvested was based on the length of the angular branch [11]. Therefore, we believe that the angular branch should be harvested with the scapula when the lateral border and inferior angle of the scapula are used for reconstructive surgery.

Variations and morphometrics

Wagner and Bayles reported four vascular patterns of the angular branch of the thoracodorsal artery. However, there have been claims that the artery can have additional variations [1,4,12]. The position of the angular branch is most peering into the muscular 5-7 cm above the tip of the bone, so sometimes it is too short for harvesting and is no longer suitable for grafting [13,14].

Though the angular artery has been found to be 40-50 mm, Seitz et al. found it to be longer [12,15]. The angular branch arises as the terminal part of the subscapular artery and forms part of the scapular anastomosis, connecting with branches around the scapula. Based on our findings, the angular branch can be shorter and enter the lateral border instead of the inferior angle. In cases of a thick inferior-angle cortical bone, the angular branch might reach the inferior angle.

Angular branch and lateral border of the scapula in reconstructive surgery

The angular branch of the thoracodorsal artery is important in reconstructive surgeries, particularly those involving the head, neck, and maxillofacial regions [16]. Its reliable vascular supply to the lateral border and inferior angle of the scapula makes it a preferred source for bone grafts [15]. The robust blood supply ensures the viability of the grafted tissue, promoting healing and integration into the recipient site. Studies have highlighted the efficacy of using the inferior angle, vascularized by the angular branch, in complex reconstructions, including mandibular and maxillary defects [3]. As the study by Choi et al. in 2015, the angular branch-based scapular tip free flap had achieved success in mandibular reconstruction [16]. Clinically, a useful corticocancellous scapula can be up to 12-14 cm long [17-21]. Our results further provided additional anatomical evidence and showed the importance of the lateral border of the scapula, which receives ample blood.

This approach is favored due to the consistency of the vascular anatomy and the relative ease of harvesting the graft. It is believed that the type of angular branch origin is the most important factor in pedicle length, suggesting that preoperative angiography is ideal for assessing the feasibility of the scapula flap [12]. Using the angular branch in free flap procedures provides a dual benefit: it offers a reliable blood supply to the bone and allows for transferring a composite flap, including skin, muscle, and bone, from the scapular region. This comprehensive approach is beneficial in large or complex defects requiring extensive reconstruction.

There are limitations to this study, including the small sample size of the cadaver study. The size and position of this artery are deep beneath the muscles, and in embalmed cadavers, it is tightly attached to the muscle and bone. However, specimens from these study collections may still serve as an important point of reference for anatomical and clinical information.

## Conclusions

We confirmed that the angular branch of the thoracodorsal artery supplies the lateral border and inferior angle of the scapula. This fundamental anatomical knowledge will help plan future reconstructive surgeries. Innovation and refinement of surgical techniques for harvesting and utilizing grafts based on the angular branch are also crucial. Developing new surgical approaches and tools through collaboration between anatomists and surgeons, followed by clinical validation, will improve these procedures' efficiency and success rates, minimizing complications and enhancing patient recovery​. These focused research efforts will significantly contribute to the understanding and utilization of the angular branch in clinical practice, ultimately advancing the field of reconstructive surgery and improving patient outcomes.
